# Learning Who Knows What: Children Adjust Their Inquiry to Gather Information from Others

**DOI:** 10.3389/fpsyg.2016.00951

**Published:** 2016-06-27

**Authors:** Candice M. Mills, Asheley R. Landrum

**Affiliations:** ^1^School of Behavioral and Brain Sciences, The University of Texas at Dallas, RichardsonTX, USA; ^2^Annenberg Public Policy Center, University of Pennsylvania, PhiladelphiaPA, USA

**Keywords:** social cognition, selective trust, questions, information seeking, childhood, inquiry, cognitive development

## Abstract

The current research focuses on how children’s inquiry may be affected by how they learn about which sources are likely to provide accurate, helpful information. Four- and 5-year-olds (*N* = 188) were tasked with asking two different puppet informants – one knowledgeable and one not knowledgeable – questions to determine which of four pictures was inside of a set of boxes. Before beginning the task, children learned about the knowledge status of the two informants in one of three learning conditions: (a) by witnessing how the informants answered sample questions (i.e., show condition), (b) by being told what informants knew (i.e., tell condition), or (c) by both (i.e., show & tell condition). Five-year-olds outperformed 4-year-olds on most parts of the inquiry process. Overall, children were less certain about which informant had been most helpful when they found out that information solely via observation as compared to when they had some third-party information about the informant knowledge. However, children adjusted their questioning strategies appropriately, more frequently asking questions that served to double check the answers they were receiving in the observation only condition. In sum, children were highly resilient, adjusting their questioning strategies based on the information provided, leading to no overall differences in their accuracy of determining the contents of the boxes between the three learning conditions. Implications for learning from others are discussed.

## Introduction

Children are often confronted with problems that they cannot immediately solve on their own. At times, they may discover the right answers through exploration (e.g., Legare and Lombrozo, 2014) or simply eavesdropping on others (e.g., Mills et al., 2012). Other times, though, children must actively seek information from others by asking questions.

Past research has identified that the process of gathering information by asking questions involves at least four steps: (1) recognizing when solving a problem may require assistance from others, (2) deciding whom to question, (3) determining what to ask, and (4) deciding how much information to ask for in order to solve a given problem (see Mills and Landrum, 2014, for a review). Not surprisingly, there are developmental improvements in children’s ability to successfully use this process of inquiry to gather information: 5-year-olds tend to be more successful than 3- and 4-year-olds at identifying whom to question and at asking enough questions to narrow down the solution set to one possible correct answer (Mills et al., 2010, 2011).

The current research focuses on how inquiry is affected by the *way* children learn about which sources are most likely to provide accurate, helpful information. When faced with multiple potential sources of information, young children have to override the assumption that all sources should be accurate (Harris and Koenig, 2006; Jaswal et al., 2010; Mills, 2013), which takes considerable cognitive resources. Thus, it is often challenging for children to determine which informant is most likely to provide accurate, helpful responses to questions. Presumably, the more challenging it is to determine which informant to question, the more difficult it is to engage in successful inquiry.

Evidence to date supports this claim. For example, Mills et al. (2011) created a task during which 3- to 5-year-olds were able to direct questions to puppets (i.e., informants) in order to determine which of two or four cards was hidden inside of a box (see also Mosher and Hornsby, 1966; Chouinard, 2007; Legare et al., 2013). Before the task, children were given the opportunity to learn through experience how the informants respond to questions (with the design based on selective trust research; see Mills, 2013 for a review). In the within-subjects *ignorant* informant condition, children were introduced to one informant who answered sample questions correctly and would presumably answer questions correctly during the task (i.e., the knowledgeable informant) and one informant who verbally indicated his ignorance on sample questions (i.e., the ignorant informant) and did not provide answers to the questions. In the within-subjects *inaccurate* informant condition, a knowledgeable informant was contrasted with an informant who consistently provided inaccurate responses to sample questions but did not verbally indicate ignorance (i.e., the inaccurate informant). After the introduction, children were encouraged to ask the informants questions to determine what was inside a set of boxes. It was expected that children would have an easier time distinguishing between the helpful and unhelpful informants in the ignorant informant condition than in the inaccurate informant condition, given that discounting a source who clearly marks ignorance with paralinguistic cues and provides no answer is easier than discounting a source who does not indicate ignorance and provides an answer (e.g., Koenig and Harris, 2005). Children performed as expected, directing more questions to the knowledgeable informant and obtaining more correct answers when the knowledgeable puppet was contrasted with an ignorant puppet (i.e., the ignorant condition) as opposed to an inaccurate one (i.e., the inaccurate condition; Mills et al., 2011).

This evidence suggests that children’s success at using inquiry to gather information is influenced by how easy it is for children to determine which informant will be most helpful. Building on this finding, we ask the following question in this study: how do ways of learning about each source’s knowledge status influence the success of children’s inquiry? Indeed, there are a number of different cues that can provide information about how someone might respond to questions. For instance, a child may learn that someone is good at math from observing that person’s behavior (e.g., witnessing Courtney repeatedly solving math problems correctly) or from third-party report (e.g., hearing “Courtney is good at math”). Certainly, observing someone’s behavior on a task can help children make some inferences regarding that person’s likely future behavior on a similar task (e.g., likelihood of providing helpful answers to questions; see Landrum et al., 2015 for a theoretical framework; see Mills, 2013 for a review). Indeed, at times, first-hand observation may not lead to clear intuitions regarding future behavior. For example, preschool-aged children who witnessed short demonstrations of informants’ knowledge in training performed nowhere near ceiling when making decisions regarding which informant to trust for new information (e.g., Birch et al., 2008). Their performance suggests a level of uncertainty in whom to trust that might not be present if children are explicitly told which informant is likely to be most helpful.

Supporting this, recent research has suggested that preschool-aged children are more successful at predicting someone’s behavior and explicitly labeling which informant is most accurate when the information has been gained via third-party report (e.g., “Johnny is very nice”) as opposed to via descriptions of behavioral evidence (e.g., “Johnny helped carry a woman’s box”; Liu et al., 2007; Johnston et al., 2015). There is also evidence that being forewarned about a source’s knowledge status or intentions improves performance on related cognitive tasks. For example, adults who receive information about the possible deceptive or inaccurate nature of a source prior to being exposed to the source’s message tend to process the message more cautiously (Petty and Cacioppo, 1986), and preschoolers are much better at rejecting deceptive claims when they have been explicitly warned of a speaker’s potentially deceptive intent (Lee and Cameron, 2000). The key thread in these separate lines of research is that third-party reports regarding an informant’s knowledge status may be beneficial for children as they engage in the inquiry process. Crucially, explicitly labeling one informant as being very helpful at answering questions and another informant as not being very helpful at answering questions may save children the cognitive resources needed to decide to which source to direct their questions. As long as children believe the person providing the labels is knowledgeable and helpful (instead of deceptive; see Shafto et al., 2012; Landrum et al., 2015), then children may feel comfortable trusting that person’s labels. Moreover, it may be that the *combination* of explicit labeling followed by the opportunity to observe behaviors that match the descriptions provided in the explicit labeling leads to the best understanding of who knows what.

In order to examine how different aspects of the problem-solving process are influenced by the way in which children learn about how informants are likely to respond to questions, we used a moderately constrained problem-solving task: children were presented with four pictures that could be inside a box and were given the opportunity to ask questions to either or both of two informants in order to determine which of the pictures was inside the box. Before beginning the task, children learned about the knowledge status of the two informants in one of three *learning conditions*: a) behavioral observation of a scripted question-and-answer training session—henceforth labeled as the *show* condition, b) third-party report through an explicit labeling training session – the *tell* condition, and c) both third-party report and behavioral observation – the *show & tell* condition. Mirroring past research (Mills et al., 2011), each child solved problems with two sets of informants that they were introduced to within their learning condition: (1) a knowledgeable informant contrasted with a guesser informant who clearly marked his ignorance with paralinguistic cues before providing an inaccurate guess (the *guessing* informant condition) and (2) a knowledgeable informant contrasted with an inaccurate informant who provided an inaccurate answer without further cues (the *inaccurate* informant condition).

Consistent with past research, we hypothesized developmental differences in performance overall: we expected that 5-year-olds would outperform 4-year-olds in directing questions to the appropriate sources, in asking effective questions, and in obtaining correct answers to the problems. We also hypothesized that explicitly labeling each source’s knowledge status before children began the problem-solving task (the show & tell condition as well as the tell condition) would make it easier for children to determine which source to question than when they had to learn each informant’s knowledge status from observation alone (the show condition). Furthermore, we hypothesized that the show & tell condition would lead to the best performance, since children would have the benefit of both explicit labels and evidence from observation confirming those labels to guide their interactions during the task.

Moreover, in our view, inquiry can be challenging, and thus excessive cognitive load at one point in the process may tax resources throughout the process (Mills and Landrum, 2014). In other words, if a child is straining to figure out which informant to question, the child may also struggle to figure out *what* to ask: there may simply not be enough mental resources available for the inquiry process. Based on that perspective, we expected that learning condition would influence the rest of their performance on the task: children would ask more effectively worded questions and would accurately solve more problems for the conditions in which each source’s knowledge status is explicitly labeled, performing better for the show & tell condition and the tell condition compared to the show condition. Similarly, we also anticipated that children would perform better on all aspects of the task when a knowledgeable informant was paired with a guessing informant than when paired with an inaccurate informant. Since the guessing informant continues to express his ignorance before providing an answer throughout the study, children’s cognitive load should be markedly reduced. Finally, we expected that how children learned about the sources might influence other aspects of the problem-solving process, such as their explicit understanding of which informant was most accurate and the kinds of questioning strategies children employed to solve the problems.

## Materials and Methods

### Participants

Participants were 96 4-year-olds (*M*_age_ = 4.6, *SD* = 0.30 years; 46 female) and 92 5-year-olds (*M*_age_ = 5.4, *SD* = 0.23 years; 40 female) recruited from preschools in the greater North Dallas area. Six additional children (5 4-year-olds, 1 5-year-olds) began testing but were excluded for being unwilling to engage in the tasks (e.g., disruptive, unwilling to respond to questions). The sample was mostly white and middle class with 8% reporting that they were of Latino descent. Each child was tested two separate times in a quiet room in the preschool, each session taking about 20 to 25 min.

### Overview

This data was part of a larger study examining how children learn from others. This study was carried out in accordance with the recommendations of the Institutional Review Board of the University of Texas at Dallas. Participants and their parents provided informed consent. On 1 day of testing, children engaged in tasks designed to examine their abilities to solve problems by directing effective questions to the most appropriate informants. A second day of testing examined questions not relevant to the current paper. Children received a small toy after each day of testing along with a certificate after completing the second day of testing.

### Materials

Forty-seven different simple line drawings on small notecards were used in the study. For the first warm-up task, 7 cards were designed to prime the children to attend to the dimensions of the drawings on the cards (i.e., shape and color). For the test phase, 40 cards were used. Each trial (8 total, 4 for each of the two within-subjects informant conditions – the inaccurate informant condition and the guessing informant condition) consisted of five cards that varied along the two dimensions. One card was the target card (e.g., a green apple) that was hidden inside a box, and four cards served as the possible options for the question-asking task (e.g., a green apple, a green truck, a red apple, a red truck). Eight 3.5″ × 3.5″ × 1″ boxes were used, each containing a different target card and corresponding set of options.

Two animal puppets were used in the second warm-up task designed to encourage the children to become more comfortable asking questions (a pig and a sheep) and two ecologically matched pairs of animal puppets were used as informants for the study (a lion and a bear, and a horse and a cow).

### Design

**FIGURE 1:**
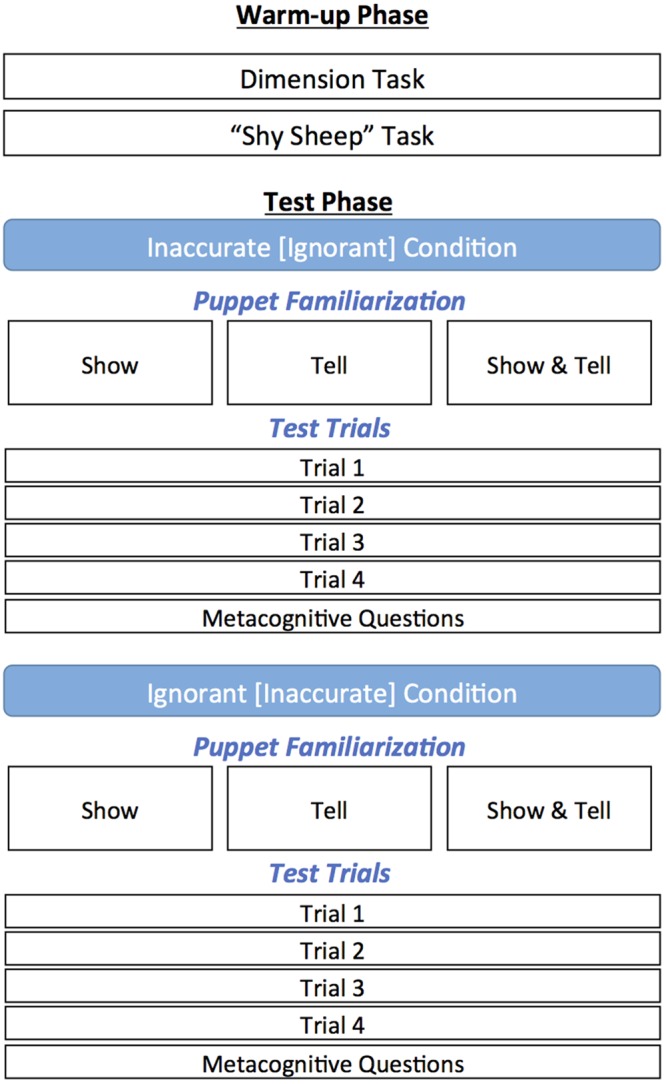
**Flowchart depicting the study design.** Participants maintained the same learning condition (puppet familiarization) throughout the study (e.g., a participant was in the Show condition for both sets of test trials).

Two experimenters conducted the study: an experimenter who interacted with the child and an assistant who monitored the stimuli, recorded the data, and served as a confederate. Each experimental session consisted of a warm-up phase and a test phase (see **Figure 1**). The test phase utilized a split plot experimental design, S(A)xB, where learning condition (show, tell, show & tell) was a between-subjects manipulation and informant condition (inaccurate informant, ignorant informant) was a within-subjects manipulation. We describe this design in more detail in Section “Test Phase”.

### Warm-Up Phase

The children participated in two warm-up tasks designed to prepare the participants for the test phase. The first task was a game designed to help participants think about the different dimensions of the line drawings on the cards (e.g., color, shape, function). The purpose of the task was to prepare children to attend to the characteristics of each line drawing so that they would be armed with more potential questions to ask later in the test trials. This task contained three parts. In the first part, children were asked to describe one card (e.g., a line drawing of a green frog). In the second, children were asked to describe a pair of cards that varied along one dimension (e.g., an orange key, an orange spoon). In the third, children were asked to describe four cards that varied along two dimensions (e.g., a yellow hat, a red hat, a yellow camera, and a red camera). Children were first encouraged to respond with as much information about the pictures on cards as they could think of. Then, children were encouraged to think about the different dimensions of the cards that they had not mentioned. For the two-card part and the four-card part, children were encouraged to think about the similarities and differences between the cards, if they did not spontaneously provide this information.

For the second warm-up task, children were introduced to a situation in which a sheep puppet wanted to ask a pig puppet questions, but was too shy (i.e., *the shy sheep task*). This task had been used in prior research to encourage children to elicit different kinds of questions (see Crain and Thornton, 1998). Each child was encouraged to put the sheep puppet on his or her hand. Then, the child was told the following, “Sheep is very shy. He wants to ask Pig some questions, but he’s so shy he can’t think of what to say. Let’s help him! I’ll tell you what Sheep would like to ask, and you make him ask Pig the question.” The experimenters matched the puppet gender to the gender of the child. The child was prompted to ask three questions, designed so that one would elicit a yes response (Does Pig like ice cream?), one would elicit a no response (is Pig’s favorite color pink?), and one was open ended (What is Pig’s favorite toy?). For instance, the experimenter said, “Sheep wants to know if Pig likes ice cream. Can you make Sheep ask Pig if he likes ice cream?” If children had difficulty formulating a question or were not sure what to say, the confederate would provide a suggestion, such as “how about saying, ‘hey Pig, do you like ice cream?”’ The goal of this task was to help the child become more comfortable asking questions and interacting with the puppets, and to hear both yes and no answers.

### Test Phase

The test phase included two within-subjects informant conditions: an *inaccurate informant condition*, in which a knowledgeable puppet was contrasted with an inaccurate puppet who always gave inaccurate, but plausible, answers, and a *guessing informant condition*, in which a knowledgeable puppet was contrasted with a guesser puppet who verbally indicated a lack of certainty (e.g., “hmm, I’m not sure. I’ll guess…”) and then provided an inaccurate, but plausible, response. For each informant condition, children first learned about the puppets (i.e., puppet familiarization) before continuing to the test trials. Each informant condition included four test trials (i.e., four boxes). The puppet pairs, the stimulus sets, and the order of the within-subjects informant conditions were counterbalanced between-subjects.

The test phase also included three between-subjects learning conditions: a *tell* condition, in which children heard a label of how each informant was likely to answer questions, a *show* condition, in which children watched a demonstration for how each informant was likely to answer questions, and a *show & tell* condition, in which children experienced both. These are described in more detail below.

#### Puppet Familiarization

For each of the two within-subjects informant conditions, children experienced one puppet familiarization task and a set of test trials (see **Figure 1**). The familiarization task was designed so that children could meet the puppets and get a sense for how the puppets answer questions without biasing the children toward the specific questions used for solving the problems in the test trials.

After the warm-up phase, children were introduced to the first pair of puppets who would help them figure out which of four pictures was inside the box. Children were introduced to the two puppets by name (e.g., Lion and Bear) and were encouraged to say hello. There were three different between-subject learning conditions for the puppet familiarization phase.

In the tell condition, the participants learned of the puppets’ knowledge statuses by the experimenter’s explicit labeling of the puppets. For the knowledgeable puppet, the experimenter said, “I’ve heard that [Puppet name] is someone who knows a lot and can give us right answers.” For the inaccurate puppet, the experimenter said, “I’ve heard that [Puppet name] is someone who doesn’t know much and gives us wrong answers.” For the guesser puppet, the experimenter said, “I hear that [Puppet name] is someone who doesn’t know much, and so he just takes a guess.”

In the show condition, the children learned about the puppets’ knowledge statuses through experience watching the puppets answer questions. This included two tasks: a question-and-answer task and a guessing game. For the question-and-answer task, the experimenter asked each puppet a question (e.g., what is toothpaste used for?) and the puppets responded according to their knowledge status (e.g., accurate: “to brush your teeth with so they are nice and clean!”; [guesser]/inaccurate: “[hmm, I’m not sure. I’ll guess] to have as a snack before bedtime!”). Each puppet was asked the same question and the process was repeated with a second question (e.g., why do people carry backpacks). Which puppet was questioned first was counterbalanced as well as which question was asked first. Importantly, children heard both puppets respond to each question. For the guessing game, the child was told that they (the child, the experimenter, and the puppets) would hide one of two items (e.g., a ball or a stuffed toy) inside of a paper bag, and the confederate had to guess which one it is by asking the puppets questions. The confederate was told to cover her eyes while the child picked which of the two items to place inside the bag. In the first trial, the confederate asked the inaccurate/guesser puppet a question about what is inside of the bag (e.g., is the thing in the bag blue?). After the inaccurate/guesser puppet provided a wrong answer, the confederate guessed the wrong item. The confederate then asked the experimenter for another try. In the second trial, the confederate asked the accurate puppet a question and then guessed the right item.

In the show & tell condition, children learned about the puppets’ knowledge statuses by both hearing the experimenter’s explicit labeling of the puppets and the question-and-answer demonstration and guessing game. The explicit labeling always preceded the question-and-answer demonstration and guessing game.

#### Test Trials

As previously stated, there were four test trials in each within-subjects informant condition, for a total of eight trials. At the beginning of the test trials for each condition, a box and four pictures for the first trial of the set were placed on the table, and children were reminded, “One of these four pictures is inside of the box. You can ask our puppet friends questions to figure out which one is inside. You can ask our puppet friends questions about what the thing in the box looks like, sounds like, feels like, does, or any other question about these pictures that will help you figure out which one is inside. But, you cannot ask us which picture is the one inside the box. When you think you’ve figured out what is inside the box, you can guess. Are you ready? Which one of my puppet friends do you want to ask first?”

The experimenter had prepared responses to questions developed from methods used in previous research (Mills et al., 2011) and through piloting the current study. In general, the experimenter encouraged the child to ask questions about the item inside the box and redirected ineffective questions. See **Table 1** for specific example questions and responses for Experiment 1.

**Table 1:** Codes for the types of questions children asked.

Types of questions	Explanation and/or example question(s)
**Effective questions**	
Function/behavior	*What does it do?*
Property	*What color is it?; What shape is it?*
A part of the whole	*Does it have leaves?*
**Ineffective questions**	
Repetitive	The information has already been given by that puppet
Vague	Not clear what the child is asking
Does not distinguish	For example, the child asks *is it blue?* when all of the options are blue.
“Why” Wording	*Why is that apple green?*
Item	*What’s in the box?*
Off-Task	*Is your dad a fireman?*
Irrelevant	For example, the child asks *is it blue?* when none of the options are blue.

Effective questions directed to either puppet informant were answered by that informant according to its knowledge status: the knowledgeable puppet gave the correct answer, the inaccurate puppet gave an inaccurate answer that was plausible based on the options (e.g., if the child asked what color the thing in the box was, the puppet would respond with an incorrect color that was on one of the options but not the correct one), and the guesser one always expressed uncertainty followed by an inaccurate but plausible answer (e.g., “Hmm…I’m not sure. I will guess…”). After each question and answer exchange, the experimenter asked the child if he or she wanted to ask another question or take a guess, reminding the child that the point of the game was to figure out what was inside the box.

At the end of each within-subjects informant condition, children answered several metacognitive questions aimed at testing whether children recognized/remembered the knowledge status of each puppet. First, children were asked which puppet provided more right answers. Second, they were asked to indicate their certainty (“really sure”, “a little sure”, or “not so sure”). For this item, we used a certainty scale that originally appeared in Woolley et al. (2004). Third, children were asked whether they thought that one of the puppets provided more wrong answers, and if so, which one.

After children completed one within-subjects informant condition, they were introduced to a new set of puppets and experienced the puppet familiarization task and a new set of test trials for that particular condition (see **Figure 1**).

#### Overview of Coding Scheme

Each session was recorded and then transcribed verbatim. Any information seeking statements or behaviors (i.e., questions) were identified and flagged for coding. Each question was assigned a series of codes. The first code was an “expert code” and reflected to whom the question was directed (e.g., the accurate puppet, the inaccurate puppet, the guesser puppet). The other codes reflected what type of questions children asked. The “Global Question code” categorized a question as effective (e.g., “what color is it?”), ineffective (e.g., “is your dad a fireman?”), a clarification (e.g., “what am I supposed to do again?”), or a guess (e.g., “is it the blue apple?”). For questions categorized as effective or ineffective, children received an additional “Type of Question” code. For effective questions, this code reflected what the question was about (e.g., physical properties of the object, the function of the object). For ineffective questions, the code reflected why the question was ineffective (e.g., off task, vague, not helpful in distinguishing between the options). Two coders were used and interrater reliability was 99.42%. See **Table 1** for sample questions.

## Results

### Overview

Preliminary analyses revealed no significant main effects or interactions based on the within-subjects informant condition (i.e., whether a knowledgeable informant was contrasted with an inaccurate one or a guesser).^1^ To simplify the analyses, the data were collapsed across this variable for the rest of the analyses.

The analyses are divided into four sections. The first focuses on the total number of questions asked in this experiment. The second examines children’s understanding of which informant was most knowledgeable. The third focuses on the kinds of questions asked and the strategies involved in asking questions. The final section focuses on accuracy and its correlates.

### Total Number of Questions

For each child, the number and type of questions directed to each informant were assessed. Approximately only 1 percent of questions were for clarification. Because these clarification questions were not of central issue to the study, clarification questions were excluded from the rest of the analyses.

Overall, for both 4-year-olds and 5-year-olds, the total number of questions asked during the course of the experiment was not normally distributed. A Shapiro–Wilk test of normality found that *p* < 0.005 for both age groups, with the data distribution positively skewed. For 4-year-olds, children asked as few as 0 questions^2^ and as many as 32 (*M* = 11.42, *SD* = 6.79). For 5-year-olds, this range stretched a bit farther, with as few as 0 and as many as 40 (*M* = 13.34, *SD* = 6.86). Very few children asked more than 32 questions, though, with most hovering around the mean. Thus, non-parametric tests were used to further examine this data.

To examine age group differences, a Mann–Whitney *U* test was conducted, revealing that 5-year-old trended toward asking more questions than 4-year-olds, (*M_5_* = 13.34, *SD_5_* = 6.86 compared to *M_4_* = 11.41, *SD_4_* = 6.79), *U* = 3739, *p* = 0.069.

To examine learning condition differences (collapsed across age groups^3^), a Kruskal–Wallis non-parametric test was conducted, revealing a slight trend toward a significant difference in the total number of questions asked between the learning conditions, *X*^2^(2) = 4.56, *p* = 0.10. A Mann–Whitney *U* test revealed that children asked more questions in the show condition than the tell condition, *M*_show_ = 13.45, *SD*_show_ = 7.20, *M*_tell_ = 11.00, *SD*_tell_ = 7.04; *U* = 1527, *p* = 0.049. There were no other significant differences. The number of questions for the show & tell condition fell between these two values but closer to the number asked in the show condition, *M*_show&tell_ = 12.69, *SD*_show&tell_ = 6.25.

### Understanding Which Informant Was Most Knowledgeable

#### Directing Questions to the Most Knowledgeable Informant

Descriptively, the majority of the questions were directed toward the knowledgeable informant over the other informant. Overall, children directed approximately 70% of their questions to the knowledgeable informant at greater than chance levels, *t*(179) = 11.32, *p* < 0.001, regardless of age or learning condition (4-year-olds: *M*_tell_ = 67.6%, *SD*_tell_ = 20.3%, *M*_show_ = 71.1%, *SD*_show_ = 20.2%, *M*_show&tell_ = 63.5%, *SD*_show&tell_ = 21.8%; 5-year-olds: *M*_tell_ = 70.3%, *SD*_tell_ = 25.6%, *M*_show_ = 69.3%, *SD*_show_ = 19.1%, *M*_show&tell_ = 74.0%, *SD*_show&tell_ = 18.7%).

This appears higher than the percent of questions directed to the knowledgeable informant in other research contrasting knowledgeable and less knowledgeable informants (∼60%), although it is difficult to compare the performance completely due to task differences (Mills et al., 2011). We will return to this point in the discussion.

There are two ways to analyze to what extent children directed questions toward the most knowledgeable informant. In one way, we can examine the *total* number of questions that children directed toward the most knowledgeable informant. Presumably, the more questions children direct toward the knowledgeable source the more likely they are to successfully solve the task. However, examining the total number of questions directed toward the accurate source discounts the amount of distracting information that children may have been receiving from questions asked to the unhelpful source. Moreover, this method may more heavily weigh data from children who asked a large number of questions. In a second way, we can examine the *percentage* of questions directed to the knowledgeable informant. This method takes into account the extent to which children preferred the accurate informant over the other informant, but it may skew interpretation when children were efficient with their question-asking and thus asked few questions overall, leading to more volatile percentages. That is, efficient question-askers’ percentage scores would be more heavily influenced by directing one question to a different source than the scores of children who asked a lot of questions. Thus, we look at both types in our analyses below.

Neither the number nor the percentage of questions directed to the knowledgeable informant over the other informant was normally distributed (Shapiro–Wilk test, *p*s < 0.005). Instead, the data distributions were negatively skewed, such that most children were directing most of their questions to the knowledgeable informant over the other informant. Thus, first, to examine age group differences, we conducted a Mann–Whitney *U* test on the *percentage* of questions directed to the most knowledgeable informant, which revealed no differences between 4- and 5-year-olds (67% compared to 71%), *U* = 3689, *p* = 0.30. A Mann–Whitney *U* test on the *number* of questions directed to the most knowledgeable informant revealed a significant difference, *U* = 3501, *p* = 0.01, driven by older children directing more questions to the knowledgeable informant (likely because they asked more questions overall) than younger children (*M*_4_ = 7.78, *SD*_4_ = 5.40, *M*_5_ = 9.35, *SD*_5_ = 4.82).

Second, to examine differences based on learning condition, we conducted Kruskal–Wallis tests. There were no significant differences in the percentage of questions (or the number of questions) directed to the most knowledgeable informant between the three conditions, *X*^2^(2) = 0.09, *p* = 0.96, and *X*^2^(2) = 3.30, *p* = 0.19. Inspecting the data for each age group separately revealed similar patterns between the three conditions and significant variability (4-year-olds: *M*_tell_ = 7.15, *SD*_tell_ = 5.26, *M*_show_ = 10.19, *SD*_show_ = 5.37, *M*_show&tell_ = 7.94, *SD*_show&tell_ = 4.62; 5-year-olds: *M*_tell_ = 9.11, *SD*_tell_ = 4.88, *M*_show_ = 9.35, *SD*_show_ = 4.70, *M*_show&tell_ = 10.16, *SD*_show&tell_ = 4.51). Thus, learning condition did not appear to affect children’s ability to ask more questions to the most knowledgeable informant.

#### Explicit Recognition of Which Informant Was Most Knowledgeable

First, we examined children’s accuracy on the four metacognitive questions (i.e., which informant was most accurate and which gave wrong answers for the two within-subject conditions). Chi-square tests were used to examine age performance for the total number of correct answers (range from 0 to 4). Older children were accurate more frequently than younger children, *X*^2^(4) = 24.71, *p* < 0.001, *M*_4_= 2.70, *SD*_4_ = 1.20, *M*_5_ = 3.39, *SD*_5_ = 0.98. There were no significant differences based on learning condition, *X*^2^(8) = 6.37, *p* = 0.61.

Second, we examined children’s accuracy on the metacognitive *certainty scale* responses. For the metacognitive questions after each within-subjects condition, children were asked who they thought provided more right answers and how sure they were about this selection. Responses were converted to a 6-point scale, with six indicating certainty that the knowledgeable informant provided the most right answers and 1 indicating certainty that the inaccurate/guesser informant provided the most right answers.

**FIGURE 2:**
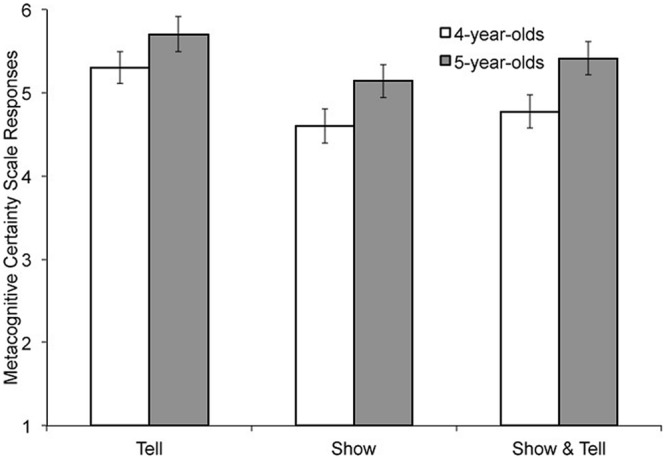
**Mean scores (possible range 1-6, where higher numbers indicate greater certainty toward the correct informant) on the metacognitive certainty scale for 4- and 5-year-olds for the three learning conditions**.

A 3 (learning condition) × 2 (age group) ANOVA on the metacognitive certainty scale responses was conducted. There was a main effect of age group, *F*(1,182) = 10.03, *p* = 0.002, $\eta_{p}^{2}$ = 0.052; older children were more confident in an accurate direction than younger children, *M*_4_ = 4.89, *SD*_4_ = 1.29, *M*_5_ = 5.42, *SD_5_* = 1.01. There was also a main effect of learning condition, *F*(2,182) = 4.86, *p* = 0.009, $\eta_{p}^{2}$ = 0.051. *Post ho*c tests with Bonferroni correction found that children were significantly more confident in the correct informant in the tell condition than in the show condition, *M*_tell_ = 5.50, *SD*_tell_ = 0.85, *M*_show_ = 4.88, *SD*_show_ = 1.36, *p* = 0.011. Performance on the show & tell condition was somewhere between these two conditions, *M*_show&tell_ = 5.09, *SD*_show&tell_ = 1.24. The interaction between learning condition and age group was not significant, *F*(2,182) = 0.17, *p* = 0.85. See **Figure 2**.

### Understanding What and How Much to Ask

#### Asking Effective Questions

The vast majority of children’s questions were effective (∼90%) rather than ineffective. This appears to mirror past work using a similar paradigm (∼87%; see Mills et al., 2011). The most common effective question by far was about a property of the item such as color or shape (75% of effective questions), although children also sometimes asked about the item’s function (22% of effective questions). Although ineffective questions were rare, the most common ineffective questions were either a “why” question that did not help children gather information to solve the problem (e.g., “why is that cat brown?”; 35% of ineffective questions) or a direct question regarding which item was inside the box, which we had explicitly stated could not be answered (e.g., “what’s in the box?”; 35% of ineffective questions).

To move forward with analyses, we first calculated the percentage of children’s effective questions out of the total number of questions. Both the number and the percentage of effective questions were not normally distributed (Shapiro–Wilks, both *p*s < 0.05). The data distribution was negatively skewed, such that most children asked many more effective questions than ineffective ones.

Thus, first, to examine age differences, we conducted a Mann–Whitney *U* test, which revealed that older children provided a greater percentage of effective questions than younger children (94.3% compared to 88.5%), *U* = 3416, *p* = 0.051 as well as a greater number, *U* = 3389, *p* = 0.006.

Second, to examine differences based on learning condition, we conducted Kruskal–Wallis tests. There were no significant differences in the percentage or number of effective questions between the three conditions, *X*^2^(2) = 2.16, *p* = 0.34 and *X*^2^(2) = 3.67, *p* = 0.16. Inspecting the data for each age group separately revealed similar patterns between the three conditions (4-year-olds: *M*_tell_ = 9.33, *SD*_tell_ = 5.80, *M*_show_ = 12.38, *SD*_show_ = 6.76, *M*_show&tell_ = 10.32, *SD*_show&tell_ = 4.45; 5-year-olds: *M*_tell_ = 11.86, *SD*_tell_ = 6.04, *M*_show_ = 12.71, *SD*_show_ = 5.16, *M*_show&tell_ = 13.13, *SD*_show&tell_ = 5.71). Thus, learning condition did not appear to affect children’s ability to ask effective questions.

#### Strategy Usage

At the level of each trial, we coded whether children engaged in some kind of confirmation strategy: asking parallel questions to the same puppet (e.g., first asking if it was shaped like one possible shape, an umbrella, then asking the *same* puppet if it was shaped like the other possible shape, a butterfly), asking the same puppet the same question more than once (e.g., first asking the puppet if it was shaped like an umbrella, then asking the *same* puppet again if it was shaped like an umbrella), and asking the same question to both puppets (e.g., asking one puppet if it was shaped like a butterfly and then asking the *other* puppet if it was shaped like a butterfly). These strategies could help children confirm they had the right answer or that the two informants said different things in ways that could help them ask the right informant questions in the future.

We then calculated the number of trials overall each child engaged in a confirmation strategy (out of a possible maximum of 8). The raw number of trials children used a confirmatory strategy was very positively skewed, and a Shapiro–Wilk test of normality supported that the data was not normally distributed, *p* < 0.05. The vast majority of children never used a confirmation strategy (*N* = 106) or used them very rarely (one trial, *N* = 38; two trials, *N* = 18). Only 11 children used these strategies for 5 or more trials. Non-parametric tests revealed there were no age or learning condition differences in how many trials children used confirmatory strategies, all *p*s > 0.10.

To better understand children’s performance in light of the skewed nature of the data, we categorized children into two groups: those who had used a confirmatory strategy at some point during the study and those who had not. Eighty-two children used a confirmatory strategy at some point in the study, whereas 106 children did not. Chi-square tests examining patterns of responses for the two age groups found no differences in the percentage of children who used a confirmatory strategy at some point, *X*^2^(1) = 0.001, *p* = 0.97.

Children did, however, trend toward showing different patterns of confirmatory strategy use across the three learning conditions, *X*^2^(2) = 4.64, *p* = 0.09. Children were more likely to use a confirmatory strategy at some point during the study for the show condition as opposed to the tell condition (50% versus 33%), *X*^2^(1) = 3.78, *p* = 0.05. We believe this finding is due to children being less certain about which informant was most helpful in the show condition compared to the tell condition, thus prompting them to double check that they were asking the correct informant. There were no other differences.

#### Asking Enough Questions

For each trial, we examined children’s patterns of questions to determine whether they had asked enough questions to successfully narrow down the options so that there was only one possible correct answer. For some children who were direct in their inquiry, only a couple of questions were needed; for others, it took many more. The common characteristic, though, was that they should have been able to determine the correct answer based on the responses they had received to their questions. Therefore, we coded each trial for whether or not the child had asked enough questions to obtain the right answer. We then calculated the number of trials overall for which each child asked enough questions (out of a possible maximum of 8). The raw number of trials children asked enough questions was positively skewed (skew = 0.85), with many children never asking enough questions (*N* = 85; 45%). Beyond that, performance was U-shaped: 21 children (11%) asked enough questions for 1 trial, 14 (7%) asked enough questions for 2 trials, 12 (6%) asked enough questions for 7 trials, and 21 (11%) asked enough questions for 8 trials, with the remainder somewhere between 3 and 6 trials.

We conducted a Mann–Whitney *U* test on the number of trials for which children asked enough questions between the two age groups, which revealed that older children asked enough questions for more trials than younger children, 3.22 (*SD* = 2.40) compared to 1.68 (*SD* = 3.28), *U* = 5511.50, *p* = 0.002. More than half of 4-year-olds (52%) and a third of 5-year-olds (38%) never asked enough questions for any trial during the course of the experiment. In other words, they stopped short in their inquiry, gathering some information but not enough to eliminate all possible answers except one. A Kruskal–Wallis test revealed no differences between the three learning conditions, *X*^2^(2) = 3.36, *p* = 0.19.

To better understand children’s performance in light of the skewed nature of the data, we categorized children into two groups: those who had asked enough questions at some point during the study and those who had not. For this variable, we found a trend toward a difference between the three learning conditions, *X*^2^(2) = 4.81, *p* = 0.09. A Mann–Whitney *U* Test revealed that the show and the tell conditions were significantly different from one another (*U* = 1576, *p* = 0.047). Children were more likely to ask enough questions for at least one trial during the study in the show condition compared to the tell condition, 62% compared to 44%; performance for the show & tell condition was more similar to the show condition at 59%.

### Accuracy and Its Correlates

#### Accuracy

**FIGURE 3:**
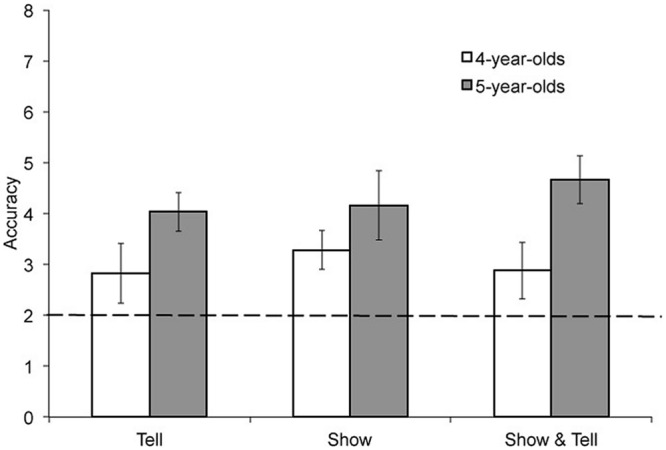
**Mean accuracy for 4- and 5-year-olds for the three learning conditions.** The dashed line represents chance performance.

The number of trials (out of a possible 8) for which children correctly identified which card was inside the box was calculated for each child. Our first set of analyses focused on the number of correct answers overall. A 3 (learning condition) × 2 (age group) ANOVA was conducted on the number of correct answers obtained. We found a significant main effect of age group, where older children were more accurate than younger children, *F*(1,182) = 19.59, *p* < 0.001, $\eta_{p}^{2}$ = 0.10. There were no effects of learning condition. See **Figure 3**.

For the number of correct answers in each condition, we also compared children’s accuracy to chance levels (i.e., since 4 possible answers for each trial, chance responding would lead to 2 correct answers out of 8 total items). Overall, children were more accurate than chance levels, *t*(187) = 10.65, *p* < 0.001. Similar findings were found for each age group and all 3 learning conditions, all *p*s < 0.001. In other words, overall, children performed consistently better than chance.

#### Relationship between Study Variables and Accuracy

Originally, we had anticipated children would obtain the most correct answers in the show & tell condition, with performance in the tell condition falling somewhere between it and the show condition. We found no evidence of that in the data. To better understand what most highly related with children’s accuracy, we examined the correlations between accuracy and the following measures: age, the number of questions directed toward the knowledgeable source (regardless of effectiveness), the number of effective questions (regardless of the source), the number of total questions, the number of trials in which children asked a confirmation strategy, the response to the metacognitive question on the 6-point scale, and the number of trials in which children asked enough questions to obtain the information needed to solve the problems. Descriptive statistics and correlations for each condition are provided in **Table 2**.

**Table 2:** Descriptive statistics and correlations between variables.

	Descriptive statistics								
	*M*	*SD*	1	2	3	4	5	6	7
(1) Accuracy	3.62	2.09	_	_	_	_	_	_	_
(2) Age	4.87	0.49	0.34^∗∗^	_	_	_	_	_	_
(3) Number of questions to knowledgeable source	8.55	5.17	0.58^∗∗^	0.19^∗∗^	_	_	_	_	_
(4) Number of effective questions	11.08	6.10	0.45^∗∗^	0.22^∗∗^	0.84^∗∗^	_	_	_	_
(5) Number of trials confirmatory strategies asked	1.05	1.74	0.16^∗^	0.04	0.63^∗∗^	0.62^∗∗^	_	_	_
(6) Number of trials enough questions asked	2.43	2.96	0.57^∗∗^	0.29^∗∗^	0.65^∗∗^	0.69^∗∗^	0.23^∗∗^	_	_
(7) Metacognitive scale rating	5.16	1.19	0.33^∗∗^	0.27^∗∗^	0.33^∗∗^	0.26^∗∗^	0.07	0.27^∗∗^	_

*^∗^*p* < 0.05, ^∗∗^*p* < 0.01.*

In general, all of the task variables noted above correlated to some extent with accuracy. Age mattered, in that accuracy increased with age. Understanding which informant was most knowledgeable mattered, in that both the number of questions directed to the most knowledgeable informant and the explicit recognition of which informant was most knowledgeable (i.e., metacognitive question accuracy) correlated with higher accuracy. Asking effective questions mattered, in that accuracy increased with the number of effective questions asked. Use of confirmatory strategies correlated with accuracy on the task, and children who used a confirmatory strategy at some point in the study performed better than children who did not, *t*(186) = 0.264, *p* = 0.009, *M* = 4.07, *SD* = 2.23 compared to *M* = 3.27, *SD* = 1.92. Finally, children obtained more correct answers if they at some point asked enough questions on a trial during the study than if they did not, *t*(182.38) = 5.32, *p* < 0.001, and the more trials in which children asked enough questions, the more correct answers they received, consistent with prior work.

A hierarchical multiple regression analysis was conducted to examine a model for predicting accuracy. We focused on three variables of interest: age, the number of questions directed to the knowledgeable informant, and the number of trials in which enough questions were asked. The number of effective questions was not included in this analysis due to this measure having little variability (i.e., 90% of the questions were effective) and due to it correlating significantly with the number of questions asked to the knowledgeable source (*r* = 0.84). Accuracy (number of correct trials out of 8) was entered as the dependent variable. Age was entered in the first step, and then these two variables were entered in the second step: (a) the number of questions directed to the knowledgeable source, and (b) the number of trials in which enough questions were asked. The addition of the variables in the second step explained a significant percentage of the variance in accuracy beyond age, Δ*R*^2^ = 0.31, *p* < 0.001. The overall model was significant, *F*(3,183) = 47.19, *p* < 0.001. The regression standardized coefficients revealed that all three predictors were significant: age (β = 0.19, *t* = 3.35, *p* = 0.001), the number of questions asked to the most knowledgeable informant (β = 0.36, *t* = 4.95, *p* < 0.001), and the number of trials in which enough questions were asked (β = 0.28, *t* = 3.71, *p* < 0.001).

## Discussion

This study examined how children gather information from others to solve simple problems. Mirroring past research, we found a number of developmental differences in performance overall. Older children were better than younger children at knowing *whom* to question: they were more likely to explicitly recognize which informant was most accurate, and they directed a larger number of questions to the more knowledgeable informant (but not a larger percentage of questions, perhaps because older children were simply asking more questions overall). In addition, older children were better than younger children at knowing *what* to ask: they asked a greater number and percentage of effective questions, and they were more likely to ask enough questions to narrow down the options to one correct answer for each item. Thus, older children performed better than younger children on most parts of the inquiry process, which led to a higher level of accuracy overall.

Our study differs significantly from past research in that we examined how different cues regarding the knowledge status of each informant (i.e., how the informants are likely to answer questions) influence children’s ability to successfully gather information for problem solving. We had hypothesized that the combination of witnessing explicit labels for each source’s knowledge status and observing how the informants answer other questions before children began the problem-solving task (the show & tell condition) would make it easier for children to determine which source to rely on for the answers to their questions than when they had to learn each informant’s knowledge status from observation alone (the show condition). Consequently, we also expected performance for just witnessing explicit labels for each source’s knowledge status (the tell condition) to be somewhere between the show condition and the show & tell conditions. In addition, we anticipated that children would perform better when a knowledgeable informant was contrasted with one who provided inaccurate responses but indicated he was guessing (the within-subjects guessing condition) than one who did not (the within-subjects inaccurate condition). Finally, we expected that how children learned about what each informant knew would relate to better performance on other aspects of the task (e.g., question types and strategies used, explicit judgments regarding which informant was most knowledgeable, overall accuracy). Our results partially support these hypotheses.

The above predictions stemmed from the idea that children’s performance on different aspects of the inquiry task would be influenced by how clearly they understood which informant was most knowledgeable. Contrary to our expectations, the within-subjects manipulation (whether a knowledgeable puppet was contrasted with a guessing one or an inaccurate one) did not influence children’s performance. As noted at the beginning of the results section and described in Footnote 1, in both within-subjects conditions, children performed similarly well at recognizing which informant was helpful and which one was not, and no differences between these conditions were found in any other aspects of the task. In contrast, and somewhat consistent with our expectations, the between-subjects manipulation (the cues children experienced regarding what each informant knew) influenced children’s recognition of which informant was most knowledgeable. Children were similarly accurate at identifying the most helpful and least helpful informants across the three between-subjects learning conditions, but what differed across the three conditions was children’s *confidence*. Indeed, children were more certain that they knew which puppet was the accurate one in the tell condition than in the show condition, with certainty levels in the show & tell condition somewhere between.

The lower level of certainty in the show condition likely led to some of the other differences between learning conditions seen in this study, and these all revolve around the questions children asked. First, children asked a greater number of questions in the show condition compared to the tell condition. Second, children were more likely to use a confirmatory strategy at some point during the study more often in the show condition than in the tell condition. In other words, not being certain of which informant was most accurate may have led children in the show condition to ask follow-up questions at some point to make sure they were gathering correct information from the most helpful informant. This, in turn, may have led to a third main difference between the learning conditions: children were more likely to ask enough questions at some point during the experiment in the show condition than in the tell condition.

Crucially, though, none of these performance differences led to drastic differences in children’s performance on the other aspects of the task. Contrary to our expectations, we found no evidence that learning condition influenced children’s ability to direct questions to the most knowledgeable informant, to generate effective questions, or to solve the problems accurately.

So why did learning condition not have a greater impact on performance? One explanation is that, overall, children were quite successful at determining which informant was most accurate. During the study, children directed over 70% of their questions to the more knowledgeable informant, a rate higher than seen in past studies (Mills et al., 2011). This better performance is likely due to differences in how children learned about the informants. In past work, the training involved puppets answering two sets of why questions that were unrelated to the problem-solving task (Mills et al., 2011). In the present work, the training was much more extensive, with puppets in the show and the show & tell conditions responding to two general questions as well as two questions specific to helping identify a hidden object – a task related to the goal of the test problems. This training provided more direct information regarding how the informants might respond on the task than the training in past work, which led to better selection of the most knowledgeable informant overall and less sensitivity to the informant contrasts (i.e., knowledgeable versus inaccurate and knowledgeable versus guesser).

Generally speaking, then, the training for all three learning conditions gave children a solid foundation for which informant would be most likely to provide correct answers to their questions. The main difference was that children were slightly less certain about who would be most helpful when each informant was not explicitly labeled. But children proved to be surprisingly adept at adjusting their strategies to determine which informant was most helpful. The show & tell condition – where children received both explicit labels for how the informants tend to answer questions and witnessed the informants actually answer questions – did not provide a boost to children’s understanding of who knew what. In the end, most children appeared to figure out to whom to direct their questions, and they reported being either a “little sure” or “very sure” which informant had been most helpful.

Another explanation for why learning condition did not have a greater impact on performance relates to where children struggled in this task—in gathering *enough* information. Overall, children were generally successful at knowing whom to question and at generating effective questions. Our regression analyses showed that, beyond age, accuracy was clearly linked to both (1) asking a larger number of questions to the knowledgeable informant and (2) to having more trials in which enough effective questions were asked to theoretically narrow down the possible options to one correct answer. In other words, success on this task was highly linked to gathering enough information from a knowledgeable source. Almost half of the children never asked enough questions on any trial to narrow down the options so that there was only one possible correct answer. Consequently, their performance suffered: on average, children obtained correct answers at greater than chance levels, but not drastically so. Older children performed better than younger children, but they were still correct around half of the time. The learning conditions were designed to influence performance at the very beginning of the inquiry process and trickle down throughout the task, but knowing whom to question was not enough to help children persist through the end in gathering enough information.

Notably, there was huge variability in performance on many aspects of the task, from how many questions were asked to how likely children were to ask enough questions to determine with certainty the correct answer. At least in the current study, it appears that the differences between children in their approaches to this task outweighed the effects of learning condition. To better understand reasons for differences in performance, ongoing work in our laboratory examines individual differences that may contribute to the ability to successfully use inquiry to solve problems (e.g., verbal skills, working memory skills). Similarly, related work has demonstrated that children’s curiosity relates to their question-seeking behavior (e.g., Jirout, 2011). Moreover, there are significant individual differences in how much children are interested in closing “information gaps” (Jirout and Klahr, 2012); thus, perhaps some children are more interested than others in gathering enough information to solve problems with certainty. More recent research has demonstrated that temperament plays a role in selective trust, with extroverted 3-year-olds being more likely than introverted ones to accurately gather information from appropriate sources (Canfield et al., 2015). Future research can examine these issues.

In sum, these results are consistent with the idea that how preschool-aged children learn about what informants know influences how they engage in the inquiry process. Our evidence suggests that labeling some sources as helpful may be useful in some circumstances, particularly when it is hard to determine which informant may provide helpful answers on one’s own. But even in the face of uncertainty, children rise to the challenge, adjusting their strategies to help them more effectively gather information.

## Author Contributions

CM initially developed this study, with both authors contributing to design, data analysis, and interpretation of the results. AL was in charge of data collection. AL drafted the method and references section; CM drafted the other sections. Both authors read and corrected draft versions. Both authors approved the final version.

## Conflict of Interest Statement

The authors declare that the research was conducted in the absence of any commercial or financial relationships that could be construed as a potential conflict of interest.

## Footnotes

1 We believe there are at least two reasons explaining this lack of difference between the two within-subjects conditions. First, past studies have found that children’s decisions regarding whom to question depend on the circumstances under which informants provide responses. When a guessing informant provided no information or an impossible answer, children learned quickly not to trust that source. In contrast, when a guessing informant and an inaccurate informant both provided plausible answers, older preschool-age children are less sensitive to whether or not the informant indicates uncertainty (see Mills et al., 2011). Our design is consistent with this latter finding. Second, in this particular study, children may have had so much experience with the informants through training that they did not receive any added boost from having the guesser informant mark his uncertainty before providing an answer. The high performance on determining which informant to question, described below, supports this idea. We also address this issue more generally in the discussion.

2 Children who asked 0 questions still participated in the study as long as they were willing to guess which picture they thought was in the box. The vast majority of children – 94% of 4-year-olds and 98% of 5-year-olds – asked at least one question.

3 For all Kruskal–Wallis tests, we also examined the data for each age group separately. No statistically significant differences were found between the three learning conditions for any Kruskal–Wallis test.
